# On Alcohol and Its Relations to Man

**Published:** 1866-07

**Authors:** 


ON ALCOHOL AND ITS RELATIONS TO MAN.

This paper on Alcohol and its Relations to Man, by Dr. Gerard F. Morgan, of Maryland, was presented and in part read by Dr. Dunbar, member of the special Committee with Dr. Morgan to present a report upon this subject.

After some discussion, it was, on motion, voted, that the report be presented by the Committee on the subject be referred to a new committee, to report at the next session.

Dr. Dunbar, of Maryland, Dr. T. Antisell, D.C., and Dr. N. S. Davis, Ill., were nominated, and the request referred to the Association.

Adjourned *sine die.*

